# Exploring the potential of cold plasma therapy in treating bacterial infections in veterinary medicine: opportunities and challenges

**DOI:** 10.3389/fvets.2023.1240596

**Published:** 2023-09-01

**Authors:** Parvin Mohseni, Abozar Ghorbani, Niloofar Fariborzi

**Affiliations:** ^1^Department of Pathobiology, Faculty of Veterinary Medicine, Shahid Bahonar University of Kerman, Kerman, Iran; ^2^Nuclear Agriculture Research School, Nuclear Science and Technology Research Institute (NSTRI), Karaj, Iran; ^3^Department of Biology and Control of Diseases Vector, School of Health, Shiraz University of Medical Sciences, Shiraz, Iran

**Keywords:** cold plasma therapy, bacterial infections, veterinary medicine, antibiotic resistance, wound healing

## Abstract

Cold plasma therapy is a novel approach that has shown significant promise in treating bacterial infections in veterinary medicine. Cold plasma possesses the potential to eliminate various bacteria, including those that are resistant to antibiotics, which renders it a desirable substitute for traditional antibiotics. Furthermore, it can enhance the immune system and facilitate the process of wound healing. However, there are some challenges associated with the use of cold plasma in veterinary medicine, such as achieving consistent and uniform exposure to the affected area, determining optimal treatment conditions, and evaluating the long-term impact on animal health. This paper explores the potential of cold plasma therapy in veterinary medicine for managing bacterial diseases, including respiratory infections, skin infections, and wound infections such as *Clostridium botulinum*, *Clostridium perfringens*, *Bacillus cereus*, and *Bacillus subtilis*. It also shows the opportunities and challenges associated with its use. In conclusion, the paper highlights the promising potential of utilizing cold plasma in veterinary medicine. However, to gain a comprehensive understanding of its benefits and limitations, further research is required. Future studies should concentrate on refining treatment protocols and assessing the long-term effects of cold plasma therapy on bacterial infections and the overall health of animals.

## 1. Introduction

In the realm of veterinary medicine, bacterial infections stand as formidable adversaries, casting a long shadow of economic losses and endangering the health of livestock and companion animals alike. This review embarks on a journey to explore innovative strategies in the fight against these infections, with a particular focus on cold plasma (CP) technology. To illuminate the path forward, let us first cast a wider net, acknowledging the diverse landscapes in which CP’s potential can flourish.

Bacterial infections, triggered by a multitude of pathogenic species, afflict animals across the spectrum, from livestock to beloved pets. Among the bacterial protagonists are *Campylobacter* spp., *Salmonella* spp., *Staphylococcus* spp., *Streptococcus* spp., *Pseudomonas aeruginosa*, *Escherichia coli*, *Bacillus* spp., and *Actinobacillus* spp. (1). These microbes have etched their presence within the animal kingdom, causing ailments that resonate through various species.

For instance, *Campylobacter* spp., a group of gram-negative bacteria, orchestrate gastrointestinal turmoil in a wide array of domesticated animals, spanning cattle, chickens, turkeys, pigs, sheep, dogs, and cats (1). Similarly, the shadow of *Salmonella* spp. looms large, casting its influence over humans and a menagerie of animals, including poultry, pigs, cattle, horses, cats, and dogs (2) Staphylococcus species establish their dominion on skin and mucous membranes, ushering in mastitis among dairy cows, a condition that exacts significant tolls on the dairy industry. Notably, *Staphylococcus aureus, E. coli*, and various streptococcal species, such as *Streptococcus uberis* and *Streptococcus agalactiae* (3), are principal actors in this costly drama (3). Meanwhile, *Staphylococcus pseudintermedius* orchestrates its narrative, inflicting skin, ear, and tissue infections in cats and dogs (4–6). A concerning twist in this tale is its zoonotic (7, 8) nature, posing a threat to human health (7, 8). Moreover, the disconcerting revelation that approximately 97.8% of methicillin-resistant *S. pseudintermedius* (MRSP) strains defy multiple antibiotics raises alarms within the veterinary realm (9, 10).

The opportunistic *P. aeruginosa*, resident on human and mammalian skin and mucous linings, casts its influence on pets like dogs and cats. It paints a canvas of diverse afflictions, ranging from skin and systemic infections (11) to ulcers, papules (12), ear infections (13), eye inflammations (14), sinus (15) issues, dental abscesses (16), and gum diseases (17).

In the case of *E. coli*, typically a benign member of animal microbiota, certain strains wield the power to unleash severe illnesses. Among them, enterotoxigenic *E. coli* (ETEC) infection emerges as the prevalent face of colibacillosis, primarily affecting young animals, notably pigs and calves (18) *Bacillus subtilis*, residing within the *Bacillus* genus, is implicated in food poisoning and associated with bovine mastitis and ovine abortion (19). Lastly, *Actinobacillus* species, gram-negative bacteria, stand accused of inciting diverse animal diseases (20).

In the face of these bacterial adversaries, veterinary medicine has long relied on the administration of broad-spectrum antibiotics. These antimicrobial agents, ubiquitous in procedures such as umbilical cord cutting, tail docking, vaccination courses, and castration, seek to mitigate the risk of infection by confronting stress and potential pathogens (21) in animals. However, a shadow looms over this practice in the form of antimicrobial resistance (AMR).

AMR, a global crisis recognized by the World Health Organization, extends its reach not only to human health but also to animals, domestic and wild, and the environment (22). The misuse and overuse of antimicrobial drugs in animal feed facilitate the emergence and dissemination of resistant bacterial strains (23, 24). This alarming trend has birthed a new era, characterized by the ascent of multi-drug resistant (MDR) pathogens and the prevalence of biofilms in food sources (22).

The battlefront of AMR primarily revolves around gram-negative pathogens and their production of β-lactamases, undermining the efficacy of β-lactam antibiotics, essential for treating both human and animal infections (25). Furthermore, microbial communities, known as biofilms, have emerged as a key player in AMR, offering sanctuary to pathogens on various surfaces, living and non-living (26).

As the arsenal of effective antibiotics dwindles, the susceptibility of both animals and humans to infections experiences a resurgence. In the current landscape, the emergence of antibiotic-resistant pathogenic bacteria, such as *Klebsiella pneumoniae*, *Acinetobacter baumannii*, and *P. aeruginosa*, paints a concerning picture (27). This escalating dilemma underscores the need for urgent action. The cunning propensity of these pathogens to form biofilms further compounds the challenge, intensifying concerns about food safety and the proliferation of antibiotic resistance (28). This complex issue demands a comprehensive approach, calling upon the scientific community to seek innovative solutions. In this critical juncture of combating MDR pathogens and biofilms, cold plasma (CP) technology emerges as a beacon of hope (29, 30).

## 2. Understanding cold plasma: its composition, generation, and applications

### 2.1. What is the plasma and cold plasma?

Often referred to as the fourth state of matter as described by Fitzpatrick (31), plasma undergoes a series of transformations with escalating energy levels in a substance. Its progression spans from solid to liquid, then to gas, culminating in a distinct ionized gas form as observed by Tabares and Junkar (32). Furthermore, plasma can be categorized based on the circumstances of its production either as low-atmospheric or high-pressure and it can also be classified into thermal or non-thermal plasmas, as discussed by Murphy and Uhrlandt (33). Additionally, non-thermal plasma forms such as CP, cold atmospheric plasma (CAP), and atmospheric pressure plasma (APP) are all linked with low-temperature plasma generation, though they exhibit slight differences in terms of their properties and uses. CP denotes a partially ionized gas with ion and electron temperatures significantly lower than those observed in traditional high-temperature plasmas. This term is often associated with plasmas that function at or near room temperature or moderate temperatures. The designation “cold” does not necessarily imply that the plasma is ice-cold; instead, it conveys that the electrons and ions are not in thermodynamic equilibrium with the gas temperature.

CAP is a specific form of CP that operates at, or close to, room temperature and at atmospheric pressure. This suggests that the plasma is formed under ordinary conditions, simplifying its handling and making it suitable for a variety of uses. CAPs are often non-equilibrium plasmas that contain different reactive species like ions, radicals, and excited molecules, which makes them particularly valuable for purposes such as surface modification, sterilization, and biomedical applications. Also known as non-thermal plasma (NTP), CAP can be generated using a range of electrical discharges. These include corona discharge, micro-hollow cathode discharge, gliding arc discharge, one atmospheric uniform glow discharge, dielectric barrier discharge (DBD), atmospheric pressure plasma jet (APPJ), and plasma needle (34).

Matrix metalloproteases (MMPs) play a critical role in the breakdown of extracellular matrix components, underpinning essential physiological processes such as tissue remodeling, wound healing, inflammation, and immune responses. Given the substantial impact of MMP dysregulation on various animal diseases, including musculoskeletal disorders, skin conditions, and tissue injuries (35) exploring novel interventions becomes imperative. In this context, cold plasma therapy (CAP) emerges as a potential avenue with its unique capacity to generate reactive species and modulate cellular responses. A closer examination reveals CAP’s intriguing implications for veterinary applications, particularly it is potential to influence MMPs. CAP holds promise in diverse veterinary scenarios—accelerating wound healing through tissue remodeling, mitigating skin disorders *via* anti-inflammatory effects, impacting orthopedic conditions like osteoarthritis, aiding oral tissue regeneration for dental health, offering antimicrobial efficacy against infections, and even influencing aspects of tumor management and equine health, notably wound healing and lameness management (36). This alignment between CAP and MMPs presents a novel avenue for enhancing veterinary therapeutic approaches and warrants further exploration.

Atmospheric pressure plasma (APP) designates any plasma formed at or close to atmospheric pressure, regardless of it being “cold” or “thermal” (high-temperature). Systems utilizing APP operate under standard pressure, thereby removing the requirement for vacuum chambers, which subsequently enhances their utility for numerous industrial applications. In summary, the primary distinctions between various types of plasma are rooted in their operating temperature, pressure conditions, and the reactive species they contain.

### 2.2. Applications of cold plasma

**Figure 1:**
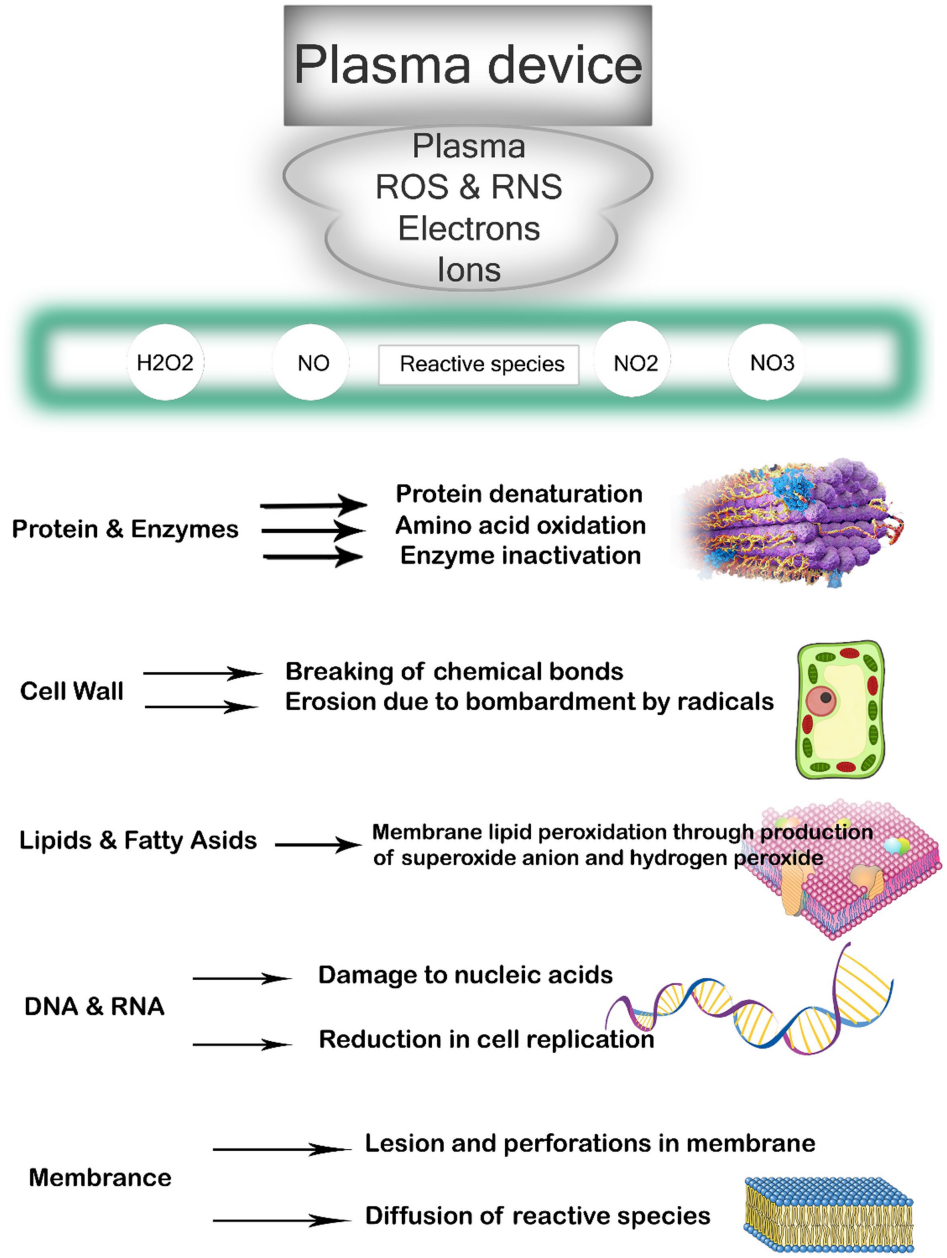
Application of cold plasma to different sections of cells.

The method employed to generate CP and the resulting composition of the plasma source plays a significant role in determining its applicability in different fields. In environmental, biological, and biomedical applications, the commonly utilized methods for CP generation are DBD and plasma jet. CP possesses a complex chemical composition, and the inactivation of microbial targets is believed to be facilitated by multiple reactive agents that act either independently or synergistically. Atmospheric air CP, in particular, contains a diverse array of reactive agents, including electrons, positive and negative ions, free radicals, stable conversion products like ozone, excited atoms and molecules, and UV photons (Figure 1) (37). Plasma sources commonly utilized produce a diverse range of reactive species, including electronically and vibrationally excited oxygen and nitrogen molecules, as well as active forms of oxygen molecules and atoms referred to as reactive oxygen species (ROS). These ROS consist of atomic oxygen, singlet oxygen, superoxide anion, and ozone (38). Reactive nitrogen species (RNS), such as atomic nitrogen, excited nitrogen, and nitric oxide, are also generated (39). In the presence of humidity, additional species such as H_2_O^+^, OH anion, OH radicals, or H_2_O_2_ can be produced. These reactive species, combined with UV radiation and charged particles, contribute to the antimicrobial properties exhibited by plasma. Among these reactive species, ozone, atomic oxygen, singlet oxygen, superoxide, peroxide, and hydroxyl radicals are believed to play a role in the inactivation of bacteria (40). The effectiveness of CP in reducing microbial growth is influenced by various factors, including environmental conditions such as temperature and relative humidity, food properties like moisture content, pH, product composition, surface properties, and surface area/volume ratio, as well as processing parameters including voltage, frequency, gas composition, flow rate, treatment time, electrode type, interelectrode gap, headspace, and exposure pattern time. Additionally, characteristics of the microorganisms themselves, such as type, strain, growth phase, and initial count, also play a role in the efficacy of CP treatment (41).

Low pH levels enhance the efficiency of CP by causing partial denaturation of protein structures and cell leakage. Moreover, the presence of water vapor in the gas phase leads to the formation of H_2_O_2_, a potent oxidizer that induces cell death by oxidizing the outer cell structure (42). CP treatment disrupts the structural integrity of microorganisms, ultimately resulting in their demise. The reactive species present in CP interact with different components of the microorganism, contributing to this effect.

Researchers confirm CP efficacy in purging surfaces and instruments from harmful organisms (43, 44). Consequently, CP stands out as a solution to decontaminate livestock environments, countering the health risks these pathogens pose to the animals (45, 46). CP short-term exposure has demonstrated efficacy in neutralizing micro-organisms within sealed spaces like airtight containers and fridges, which indicates its potential in preserving agricultural goods. This system can be applied to the storage to keep agricultural products fresh and exclusion of harmful materials from the products (47). Furthermore, CP has proven successful in neutralizing specific micromycete species and their spores, including *Cladosporium sphaerospermum*, *Aspergillus oryzae*, *Alternaria* species, and *Byssochlamys mivea* (48, 49). This fungicidal trait further underscores its value in sanitizing livestock habitats. Notably, CP can neutralize endotoxins, lipids, and prions found in micro-organisms (50–52). Additionally, CP enhances water quality on multiple fronts microbiologically, physically, and chemically by neutralizing harmful micro-organisms. Since water quality, directly and indirectly, impacts animal health and output, it is essential to monitor and maintain its quality. This management aspect falls under the infrastructure category in livestock farming (53).

## 3. Various factors affecting the inactivation efficacy of CP in bacteria

To effectively design and optimize CP devices for applications involving microbial inactivation, it is crucial to have a comprehensive understanding of how various factors influence the antimicrobial efficacy of CP. The key factors that significantly impact CP’s effectiveness include: (i) type of CP device: CP devices can be categorized into three main types: direct CP, indirect CP, and hybrid CP. (ii) Gas composition and flow rate: the composition of the gas used in CP, as well as the flow rate at which it is supplied, plays a critical role in determining its antimicrobial efficacy. (iii) Changes in source frequency, voltage, and power: alterations in the frequency, voltage, and power settings of the CP source can affect its ability to inactivate microorganisms. (iv) Changes in the safe distance for CP treatment: the distance between the CP source and the object being treated can impact the microbial inactivation efficiency. Finding the optimal safe distance is important for effective treatment. (v) CP treatment time: the duration of exposure to CP is an essential factor in determining its antimicrobial effectiveness. Longer treatment times may result in improved inactivation, but there is a need to balance treatment duration with practical considerations. (vi) Initial concentration of microorganisms: the initial concentration of microorganisms present on the treated object can influence the efficacy of CP treatment. Higher initial microbial loads may require longer or more intense CP exposure for effective inactivation. Direct CP involves one of the electrodes being part of the electrical circuit of the treated object, which can increase temperature. Examples of direct CP methods include corona discharge, DBD volume, and spark discharge. Understanding and optimizing these factors will contribute to the development of efficient CP devices for microbial inactivation applications.

Indirect CP involves the transfer of plasma generated between two electrodes to the treated object through diffusion and convection mechanisms. Various studies have noted differences in antimicrobial effectiveness between direct and indirect CP treatments. In the case of *S. aureus*, the direct CAP treatment exhibited a larger inactive zone compared to the indirect CP treatment (54). Moreover, when treating *P. aeruginosa*, both N_2_ CP and direct air CP treatments achieved a 6 log reduction in colony-forming units (CFU) within 60 s. However, in the case of indirect treatment, a 4 log CFU reduction was achieved for N_2_ CP, and a 5 log CFU reduction was achieved for air CP within the same treatment time (55). Gas plays a crucial role in CP discharge production. Typically, gases like Ar, He, air, N_2_, O_2_, or their mixtures are used for igniting plasma discharges in CP devices. Some studies have only focused on the impact of gas flow, revealing no significant effect (54, 56–58).

Limited research has addressed the impact of variations in gas flow rate on the effectiveness of CP in eliminating microbes. When the helium (He) gas flow rate was increased from 10 liters per minute (lpm) to 14 lpm, the survival count decreased from 5 × 10^8^ colony-forming units per milliliter (CFU/mL) to 6 × 10^2^ CFU/mL for *E. coli* and from 5 × 10^8^ CFU/mL to 7 × 10^3^ CFU/mL for *S. aureus*. This reduction could be attributed to an elevated concentration of active species within CP due to the increased flow rate. In a study conducted by Das et al. (59), raising the power of the cold atmospheric plasma jet (CAPJ) from 0.15 W to 1.6 W demonstrated a decreased likelihood of methicillin-resistant *S. aureus* (MRSA) biofilm formation. This outcome can be attributed to a higher flux of chemical species at greater frequencies and power, as suggested by Sharma et al. (60) and Wiegand et al. (61). In terms of the CP microjet, 13.56 MHz radiofrequency exhibited superior *E. coli* inactivation compared to the lower frequency of 50 kHz. However, similar antimicrobial effects as radiofrequency can be achieved at lower frequencies by increasing the voltage, as indicated by Kim et al. (56).

The distance between the nozzle of the CAPJ and the sample is a crucial factor that impacts the effectiveness of CP in combating microbes. Studies by Sharma et al. (62) and Shouzhe and Jinpyo (63), have shown that increasing the exposure distance leads to a decrease in the antimicrobial effect. For example, when the exposure distance was increased from 0.5 cm to 2 cm in the case of air CAPJ, Pedroni et al. (64) observed a reduction in the log colony-forming unit (CFU) reduction value from 2.5 to approximately 1.2. Therefore, the decline in CP’s antimicrobial activity with an extended exposure interval, as reported by Pedroni et al. (64) and Shouzhe and Jinpyo (63), may be attributed to a decrease in the concentration of active species during CP, as suggested by Lotfy et al. (65).

Additionally, the timing of exposure to CP also plays a significant role. When considering a fixed exposure interval in CAPJ, the duration of CP exposure required to achieve the same log reduction of *B. subtilis* is shorter in Ar + O_2_ CP compared to He + O_2_ CP. Guimin et al. (66) observed that in the case of argon CAPJ, the lethal log volume (KLV) reached 5.38 after 90 s of CP treatment for *S. aureus* and 5.36 after 60 s of CP treatment for *E. coli*.

The enhanced effectiveness of CP against microorganisms may be attributed to the interaction between active species within CP and microorganisms. This interaction intensifies with longer exposure times, resulting in a greater lethal impact. Another contributing factor is the chemical activity present in the growth environment, which ultimately influences the size of the inhibition zone. For instance, when the concentration of *E. coli* was raised from 102 CFU/mL to 105 CFU/mL, the CP treatment time increased from 30 s to 120 s. Similarly, elevating the concentration of *S. aureus* from 102 CFU/mL to 105 CFU/mL required a longer CP treatment time of 45 s to 90 s. In a study by Deng et al. (67), a 3 log CFU reduction was achieved for *B. subtilis* exposed to CP for 200 s and 360 s, respectively, when the initial concentrations were 106 CFU/mL and 109 CFU/mL. Hence, increasing the initial concentration of microorganisms necessitates a longer treatment time with CP, as indicated by Lee et al. (68).

## 4. Effect of cold plasma on bacterial structure

**Figure 2:**
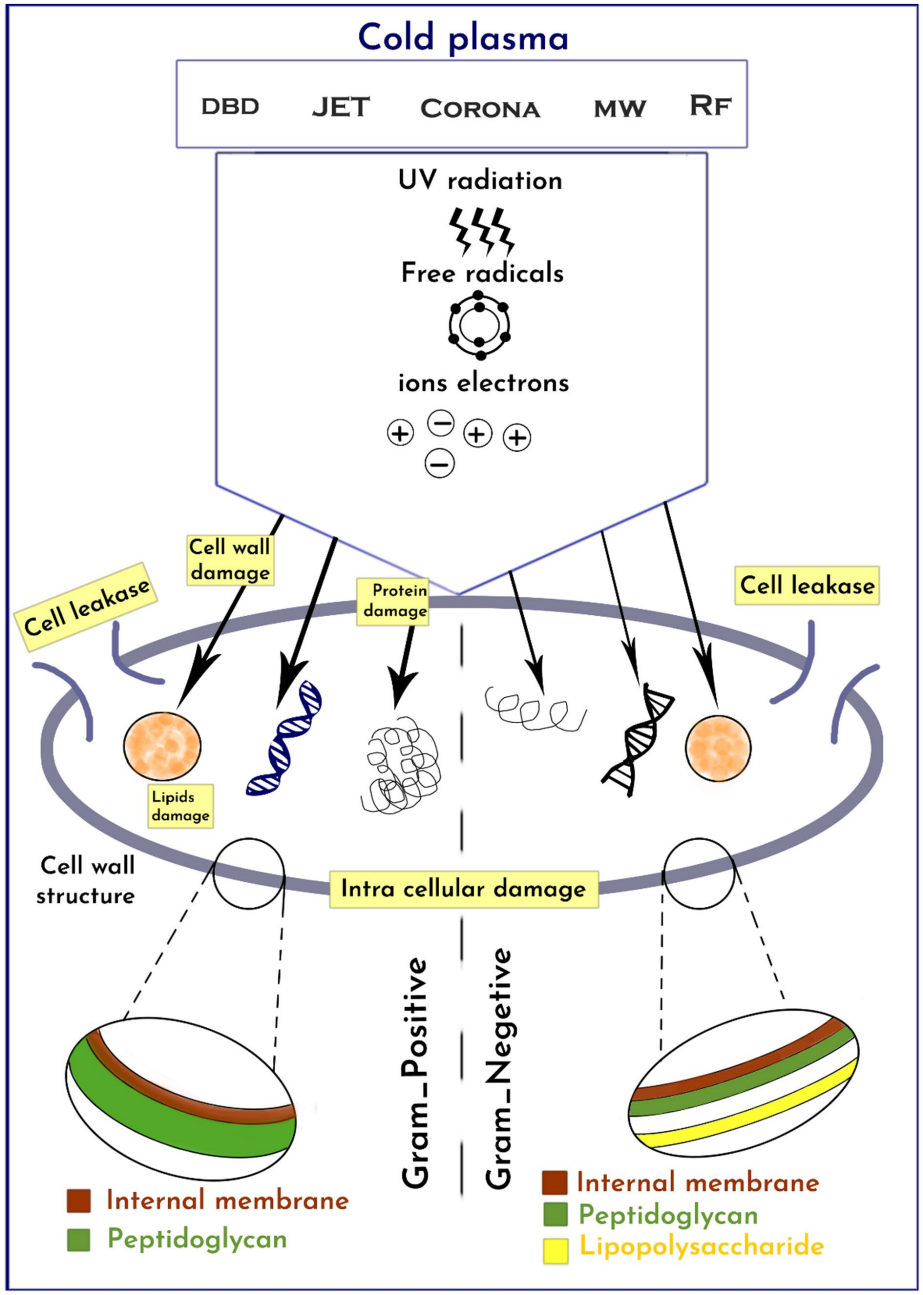
Schematics of cold plasma effect on gram-negative and positive bacteria.

CP has been shown to have various effects on bacteria, ultimately leading to cell destruction (69). The primary impacts encompass damage to the bacterial cell membrane, intracellular protein damage, and direct DNA damage, as highlighted by Niedźwiedź et al. (70). The presence of loaded particles, such as ions and electrons, gives rise to an electrostatic field that permeates the bacterial cell wall, resulting in the breakage of chemical bonds, erosion, and the formation of lesions and openings in the membranes. Additionally, these particles facilitate the entry of plasma toxic compounds into the bacterial cell, intensifying the destructive process, as stated by Olatunde et al. (71). Furthermore, proteins and bacterial DNA are directly affected by oxidative stress, as emphasized by Winter et al. (72). Reactive oxygen radicals induce significant oxidative stress, leading to lipid-oxygenation, loss of cellular cytoplasm and proteins, and oxidative DNA damage (Figure 2). This ultimately culminates in cell death when the repair mechanism becomes overwhelmed. Lastly, Winter et al. (72) illustrated in Figure 1 that before inactivation, cells undergo morphological and chemical changes.

### 4.1. Response of gram-positive and gram-negative bacteria to cold-plasma treatment

The antimicrobial effectiveness of CP varies depending on the cell wall structure of different microorganisms (59, 73). Due to their thicker peptidoglycan layer, gram-positive bacteria are less susceptible to CP compared to gram-negative bacteria (74). CP treatment causes irreversible wall damage and oxidation of GN cells due to ROS and ultraviolet (UV) radiation attacking the cell envelope, leading to the release of intracellular compounds such as proteins, DNA, and lipids. On the other hand, CP causes severe intracellular damage to GP bacteria without cell leakage. Some studies have suggested that CP is more effective against GN than GP bacteria due to the relatively thinner peptidoglycan cell wall structure of GN bacteria, which is less than 10 nm, compared to 20–80 nm for GP bacteria (73, 75).

Indeed, the susceptibility of microorganisms to CP cannot be generalized solely based on their gram characteristics, as concluded by Kim and Min (76) and Min et al. (77). The research conducted by Mai-Prochnow et al. (78) revealed that bacterial resistance to CP may be influenced by their growth conditions. For instance, *P. aeruginosa* demonstrated greater resistance to CP when in co-culture compared to a single-species biofilm. Han et al. (79) also observed distinct reactions of different bacterial strains to CP. Specifically, *E. coli* was inactivated through cell leakage due to damage to the lipopolysaccharides in the outer cell membrane and the thin peptidoglycan layers of gram-negative bacteria. On the other hand, *S. aureus*, being a gram-positive bacterium with significantly thicker peptidoglycan layers compared to *E. coli*, experienced damage to intracellular components while maintaining an intact cell wall, as demonstrated by Han et al. (79). In a study conducted by Wan et al. (80), the efficacy of high voltage atmospheric CP (HVACP) in inactivating Salmonella and its impact on egg quality were evaluated. The experiment involved treating inoculated eggs with HVACP (85 kV) in both dry air and a modified atmosphere gas environment. Direct treatment of egg surfaces under the modified atmosphere gas for 15 min resulted in a reduction of 5.53 log CFU/egg, indicating significant inactivation of Salmonella.

Another investigation by Lührmann et al. (81) examined the effect of cold atmospheric pressure argon plasma on the antibiotic sensitivity of methicillin-resistant strains of *S. aureus in vitro*. The study observed a statistically significant decrease in the growth inhibition zone. For the *S. aureus* strain SZ 148, the decrease was 6.3 mm with cefuroxime and 14.5 mm with oxacillin. Additionally, for strain 05-01825, there was a decrease of 36.8 mm with oxacillin (81). This indicates that the antibiotic sensitivity of the methicillin-resistant *S. aureus* strains was affected by cold atmospheric pressure argon plasma.

Numerous studies have been conducted in various countries to investigate the effect of CP on both gram-positive and gram-negative bacteria, as shown in Table 1.

**Table 1:** Studies related to the effect of cold plasma on gram-positive and gram-negative pathogenic bacteria in animals.

Target bacteria	CP type	Results	Reference
*S. aureus*	CP	The plate containing *S. aureus* bacteria with a 1.5 × 10^3^ CFU/mL colony count was zeroed for 10 min	(82)
*E. faecalis*	CAP	CAP reduced agar plate colony-forming units (CFU) by >7 log10 steps	(83)
*Salmonella enteritidis*	HVACP	5.53 log CFU/egg reduction after 15 min treatment of egg surfaces	(80)
*P. aeruginosa*	CAMP	Gram-negative bacteria (*P. aeruginosa* and *E. coli*) were completely killed within 60 s exposure, more susceptible than gram-positive bacteria (*S. aureus* and *S. pseudintermedius*)	(84)
*E. coli*			
*S. aureus*			
*S. pseudintermedius*			
*P. aeruginosa*	CAMP	*P. aeruginosa* completely eradicated in 120 s at 50 W	(30)
*E. coli*	HVACP	*E. coli* is inactivated through cell leakage and low-level DNA damage, while *S. aureus* is primarily inactivated by intracellular damage	(79)
*S. aureus*			
*S. aureus*	CAP	CAP generally had a negligible impact on susceptibility to antibiotics. However, in the case of two strains, there was a significant decrease in susceptibility specifically to β-lactam antibiotics	(81)
*Enterobacter aerogenes*	CP	Reductions of 4.3 ± 0.5 log CFU/surface at 3.5 kHz, 5.1 ± 0.09 log CFU/surface at 2 kHz, and 5.1 ± 0.05 log CFU/surface at 1 kHz were achieved within 2 min, 3 min, and 6 min, respectively	(85)
*E. faecalis*	CP	A 2 min exposure to CAP *in vitro* led to the complete elimination of the bacteria	(86)
*S. aureus*	LTP	The decreased growth of *S. aureus* in 30 s and decreased growth of *E. coli* exposed to 60 s with LTP	(87)
*E. coli*			
*S. aureus*	DBD	*S. aureus* cells experienced a 6.5 log reduction in CFU/mL after a 25 min treatment	(88)
*S. aureus*	PAW	*S. aureus* was significantly suppressed after a 10 min treatment, resulting in a 6 log decrease in CFU/mL	(89)
*E. coli*	HVACP	*E. coli* and *L. monocytogenes* strains were inactivated for 30 s by exposure to ACP	(90)
*Listeria monocytogenes*			
*S. aureus*	Low-pressure plasma	*B. subtilis* demonstrated a reduction within 2 min of helium plasma treatment, while *S. aureus*, *E. coli*, and *P. fluorescens* exhibited reductions after 5 min and 10 min of treatment	(91)
*E. coli*			
*Pseudomonas fluorescens*			
*B. subtilis*			
*L. monocytogenes*	CAP	Both the wild-type and knockout mutants showed similar effects after a 1 min exposure to CAP	(92)

CP, cold plasma; CFU/mL, colony-forming units/milliliter; CAP, cold atmospheric plasma; HVACP, high voltage atmospheric CP; CAMP, cold atmospheric microwave plasma; LTP, low temperature pressure; DBD, dielectric barrier discharge; PAW, plasma activated water; LPP, low-pressure plasma.

## 5. Effect of cold plasma on bacterial biofilm and MDR

Biofilms are communities of bacteria that flourish in various environments by attaching to solid surfaces and creating an extracellular matrix comprised of polysaccharides, lipids, proteins, and nucleic acids (93). These biofilms demonstrate enhanced resilience against external challenges such as antibiotics, temperature variations, and pH fluctuations. Consequently, they pose a significant hurdle in the progression of bacterial diseases in animals. Eradicating biofilms, produced by attached bacteria, presents additional complications (94). Although most research has focused on chemical bactericidal agents to combat opportunistic infections, physical treatments like CP (specific treatment) offer an alternative approach when the efficacy of chemical agents is compromised due to the natural resistance of pathogens or biofilms (95). As microorganisms can develop resistance to certain drugs over time through genetic mutation or alterations in gene sequences, the inactivation of drug-resistant microorganisms becomes increasingly challenging compared to non-resistant ones (96). Nevertheless, numerous studies have provided evidence of the effectiveness of CP in deactivating drug-resistant microorganisms, as indicated in Table 2. Furthermore, CP has shown the ability to target biofilms formed by both gram-positive and gram-negative bacteria (74, 95, 105, 110, 111). These biofilms primarily consist of water along with extracellular polysaccharides, proteins, and DNA (112). The efficacy of CP in deactivating multidrug-resistant pathogens and biofilms varies depending on the microorganisms’ structure, composition, and resistance (98, 99). The eradication of MDR superbugs, such as the “ESKAPE” pathogens, poses a major clinical challenge in the 21st century (28). Among these pathogens is *Enterococcus faecalis*, a gram-positive coccus that forms biofilms, increasing its tolerance and reducing sensitivity to antimicrobial measures (113, 114). *In vitro,* studies have demonstrated that CP exhibits antibacterial effects against both planktonic cultures and biofilms of *E. faecalis* (115).

**Table 2:** Studies related to the effect of cold plasma on MDR bacteria, biofilm and spore.

Target bacteria	CP type	Results	Reference
**Studies related to the effect of cold plasma on MDR bacteria**			
Methicillin-resistant *S. aureus* (MRSA)	CP	CP demonstrated strong antimicrobial efficacy against major skin and wound pathogens *in vitro*	(29)
Penicillum-resistant *S. aureus* (PRSA)	DBD	CP successfully inactivates *S. aureus* bacteria (wild type) as well as multidrug-resistant bacteria, including penicillin-resistant, methicillin-resistant, and gentamicin-resistant strains, to a significant extent	(97)
Methicillin-resistant *S. aureus* (MRSA)			
Gentamicin-resistant *S. aureus* (GRSA)			
Methicillin-resistant *S. aureus* (MRSA)	CP	CP treatment allowed a significant growth reduction of MRSA and MSSA	(98)
Methicillin-susceptible *S. aureus* (MSSA)			
**Studies related to the effect of cold plasma on biofilm**			
*P. aeruginosa*	CAMP	CAMP at 50 W exhibited a bactericidal effect against the ATCC strain biofilm	(30)
*E. faecalis*	CAP	In 24 h biofilms, CAP treatment resulted in a 3 log10 reduction in CFU after 5 min and a 5 log10 reduction after 10 min	(83)
*L. monocytogenes*	CP	The *E. coli* population experienced a reduction after 60 s, while the biofilms of *L. monocytogenes* and *S. aureus* underwent a reduction after 300 s	(99)
*S. aureus*			
*P. aeruginosa*	NTP	There was a rapid decline in the number of cells within the first 60 s	(100)
*E. faecalis*	CP	Inhibitory effect on *E. faecalis*	(101)
*E. faecalis*	CP	Gram-negative bacteria achieved more efficient sterilization compared to other types of bacteria	(102)
*P. aeruginosa*			
*S. aureus*	CP	*P. aeruginosa* biofilms, initially containing approximately 5.0 × 10^6^ CFU, were rapidly killed, and no bacterial survival was detected within 15 s of exposure. *S. aureus*, however, survived longer under these conditions, with no detectable growth observed after 60 s of exposure	(103)
*P. aeruginosa*			
*S. aureus*	NTP	After 1 h of exposure to MRSA, the counts were reduced by approximately 4 to 4.5 log10	(104)
*A. baumannii*	CP	The 24 h biofilm was inactivated in 540 s	(105)
*E. faecalis*	CP	Treating *E. faecalis* biofilm with plasma for 5 min resulted in a 93.1% kill rate	(106)
**Studies related to the effect of cold plasma on spore**			
*B. subtilis*	CP	Oxygen radicals have a destructive effect on both bacterial cells and endospores	(107)
*E. coli*			
*B. cereus*	CP	Cold plasma exposure effectively kills *B. cereus* vegetative cells	(108)
*B. subtilis*	CAPP	The inactivation achieved was dependent on the process gas used	(109)

CP, cold plasma; DBD, dielectric barrier discharge; CAMP, cold atmospheric microwave plasma; CAP, cold atmospheric plasma; NTP, non thermal plasma; CAPP, cold atmospheric pressure plasma.

The application of CAP treatment resulted in a significant reduction of 7 log10 steps in colony forming units (CFU) on agar plates. When 24 h *E. faecalis* biofilms were treated with CAP, a 3 log10-step reduction in CFU was observed after 5 min of treatment. In comparison, the positive controls chlorhexidine (CHX) and UVC exhibited slightly greater effectiveness than CAP in the conducted biofilm experiments. Notably, CAP treatment did not cause damage to the cytoplasmic membrane of *E. faecalis*, suggesting that membrane disruption is not the primary mechanism of action against this bacterium. Moving on to *P. aeruginosa*, an opportunistic pathogen that causes purulent inflammation in dogs’ ears and skin, its biofilms are known to contribute to antibiotic resistance and therapeutic limitations. Therefore, the antibacterial and antibiofilm properties of cold atmospheric microwave plasma against *P. aeruginosa* were studied under laboratory conditions (30). The findings indicated that complete elimination of both the ATCC strain and clinical isolates of *P. aeruginosa* was achieved within 120 s at 50 W, with the clinical isolates requiring 60 s less than the ATCC strain for complete eradication at the same power level (83). In another study by Cao et al. (101), the effectiveness of NTP (non-thermal plasma) against *E. faecalis* and *S. aureus* suspensions on agar plates, as well as *E. faecalis* biofilm on nitrate membrane filters, was assessed. Following NTP treatment, inhibition zones were observed in agar cultures, and the size of these zones increased with longer treatment durations.

DBD can be effective against MRSA strains that carry the β-lactamase gene and mecA antibiotic resistance. Through DBD, penicillin-binding proteins and regulatory factors that may affect peptidoglycan (PG) are inactivated and result in decreased β-lactamase activity, which may be due to inactivation of the β-lactamase enzyme as well as its associated gene (97). In a study by Napp et al. (98), it was observed that the effect of DBD on MRSA and MSSA is different, which could be due to the stiffer cell wall of MRSA compared to MSSA, as for certain strains by Kawai et al. (116) showed an association between thickened cell wall (MRSA) and resistance to disinfectants *in vitro* has been documented. Additional research on the impact of CP on bacterial biofilms is presented in Table 2.

## 6. Effect of cold plasma on bacterial spores

Livestock diseases often involve bacteria such as *C. botulinum*, *C. perfringens*, *B. cereus*, and *B. subtilis*. These bacterial species have developed various mechanisms to survive harsh environments, including the formation of protective spores (117). CP treatment has emerged as a promising method for inactivating bacterial spores. The primary mechanisms through which CP inactivates spores are as follows: (i) DNA damage caused by emitted UV radiation (118), (ii) erosion of the spore surface, penetration into the cortex, and damage to the spore nucleus, resulting in changes to protein structure, internal lipids, and degradation of dipicolinic acid (119), and (iii) etching of the spore coat through the action of reactive nitrogen species (RNS) (120).

Spores typically possess multi-layered outer structures that serve as the first line of defense against chemicals and physical aggression, contributing to their resistance. The external structures are primarily responsible for the spores’ resilience. Some spore species, such as *B. cereus*, possess an additional outermost layer called the exosporium, composed of proteins, lipids, and carbohydrates. Studies have shown that these extrinsic structures are targeted by CP for spore inactivation (121–123). For instance, in a study utilizing a helium atmosphere, CP treatment resulted in the complete rupture of *B. subtilis* spores, with oxidation by active plasma species identified as the primary cause of spore death (124). CP-induced damage to the spore coat leads to the release of dipicolinic acid (DPA) and increased hydration of the spore nucleus, which is a key factor in spore inactivation (125).

Multiple research studies have provided evidence that CP-generated UV photons primarily target the DNA within spores, leading to their inactivation (109, 126). Damage to the intracellular DNA can result in the death of spores. In atmospheric pressure plasma-jet (ACP-PJ), an alternative approach to increasing reactive oxygen species (ROS) production is to raise the excitation voltage, which influences energy and electron density.

In a study by Pina-Perez et al. (120), atmospheric pressure plasma-surface barrier discharge (ACP-SBD) with air as the process gas was investigated for its impact on *B. subtilis* spores. They achieved approximately 4 log inactivation of *B. subtilis* spores using a low plasma power density of 5 mW/cm^2^ and an exposure time of 7 min. Furthermore, they demonstrated that the composition of the matrix material or food substance can influence the efficiency of *B. subtilis* spore inactivation. Numerous research studies have been conducted worldwide to explore the effects of CP on bacterial spores (Table 2).

## 7. The effect of cold plasma on skin

The skin serves as a vital immune organ, playing a crucial role in protecting the body against pathogens and physical or chemical threats from the environment (127). Any damage to the integrity of the skin can result in significant impairment or even mortality. CP therapy, an innovative therapeutic approach, has been proposed for the treatment of extensive and chronic wounds. The selective eradication of severely damaged cells and the concurrent promotion of cell proliferation and migration stand as pivotal facets of this method. At the forefront of this technique is its remarkable capacity to generate stress-induced reactive oxygen species (ROS), a mechanism that enables the targeted elimination of compromised cells. Importantly, this selective action is achieved while ensuring the preservation of normal cells from harm. This fundamental capability of CP to eliminate bacteria without inducing injury to host tissue underscores a vital theme in this discussion. While the intricate details of ROS may be reserved for subsequent paragraphs, the overarching concept of CP’s ability to maintain a delicate balance between therapeutic efficacy and tissue well-being deserves prominence from the outset (128–130). In veterinary medicine, effective wound management is crucial to prevent permanent disability and economic burden.

The main components of CAP include ions, electrons, metastable, photons, and electromagnetic fields. After a reaction with environmental air, CAP forms a hierarchical group of reactive oxygen and nitrogen species (RONS) that promote increased skin tissue microcirculation, increased monocyte stimulation, increased cell migration, and stimulation of the keratinocytes and fibroblasts primarily involved in wound healing (131).

The potential influence of cold atmospheric plasma extends to the production of nitric oxide. Current understanding points to the significant part played by NO in healing wounds and regenerating tissues by managing blood vessel contraction and clotting, along with the immune system and early-stage cell death or apoptosis (132). External introduction of NO-donors—molecules that hold, release, or create nitric oxide when they come into contact with tissue—to a wound site can enhance and accelerate the healing process (133, 134). CAP may further contribute to wound recovery *via* its disinfectant properties, the stimulation of skin cell growth and movement through the activation or suppression of integrin receptors on the cell surface, or its pro-angiogenic effects (135).

A study by Amini et al. (136) showed that CAP treatment can alter the durability of inflammatory cytokines and growth factors, including IL-1, IL-8, TGF-β, TNF-α, and INF-γ, thereby facilitating healing by initiating the proliferative phase at a quicker rate. Another research piece by Arndt et al. (137) revealed that when CAP treatment was analyzed *in vitro*, it resulted in the heightened expression of crucial genes involved in the wound healing process, such as IL-6, IL-8, MCP-1, TGF-ß1, TGF-ß2. In addition, it boosted the production of collagen type I and alpha-SMA, which play significant roles in tissue repair. According to research conducted by Zhang et al. (138), the application of CP has been found to accelerate wound healing by reducing the healing time, decreasing wound size, and promoting wound contraction and re-epithelialization. Additionally, the study showed a significant increase in the protein level of smooth muscle actin, indicating improved wound healing.

Before the integration of plasma technologies into medical applications, a comprehensive evaluation of their impact on living tissues is imperative. This assessment extends to encompass an exploration of potential toxic effects resulting from plasma exposure. The significance of this pursuit lies not only in its scientific underpinnings but also in its direct implications for clinical practice. Indeed, the determination of safe and efficacious implementation avenues, free from the specter of long-term adverse effects, forms a pivotal cornerstone in advocating for the broader utilization of CP in the realm of veterinary medicine. By harmonizing the scientific and clinical dimensions, this approach resonates with the quest to establish a solid foundation for the expansion of CP’s applications within this critical domain. To evaluate the safety of plasma treatment on both intact and wounded skin, a Yorkshire pig model, which has comparable skin characteristics to humans, was utilized (139). The findings of the study indicated that plasma treatment is safe for both types of skin, even at doses higher than those needed for effective bacterial inactivation in agar or liquid media. Further research has focused on investigating the molecular and physiological consequences of CP treatment on mouse skin (115). The cells in the dermis and epidermis respond to reactive oxygen species (ROS) through redox signaling, which occurs due to environmental stress or disruptions in tissue homeostasis (140). Plasma-derived ROS induces changes in the junctional network, improves tissue oxygenation, oxidizes stratum corneum lipids, and limits the penetration of substances like the model drug curcumin (115).

In a study conducted by Lee et al. (141), the impact of CAP on skin physiological parameters and tolerance in dogs was assessed. The CAP treatment was applied to various points on the dogs’ natural skin in the groin area for durations of 30 s, 1 min, 2 min, and 4 min. Hydration, trans epidermal water loss (TEWL), and surface temperature were measured before and after the CAP treatment, with measurements taken five times, three times, and three times, respectively. The results showed that, except for mild erythema observed in the treatment area after 4 min, the majority of dogs did not exhibit significant pain responses or side effects. In another research study by Li et al. (142), the possible mechanisms of a cold atmospheric plasma jet on a rabbit wound infected with methicillin-resistant *S. aureus* (MRSA) were investigated *in vivo*. The researchers found that the treatment could regulate cytokine secretion, limit the inflammatory response and excessive cell proliferation, accelerate re-epithelialization, and ultimately promote the wound healing process while inactivating bacteria. Furthermore, the potential of atmospheric pressure plasma jet therapy for wound healing was studied using a mouse model of ear wounds in research conducted by Schmidt et al. (143). The effectiveness of CP on skin regeneration was explored in this study.

The *in vitro* experiments investigating the effect of CP on keratinocyte and fibroblast migration, specifically in the context of scratch assays, showed that CP stimulation led to significant stimulation and rapid closure of the gaps. To enhance the astringent effect of CP jets on acute wounds in mice, researchers applied microliters of distilled water to the wounds before treatment. The combined treatment of water and plasma was found to be more effective than plasma treatment alone. The water, when combined with plasma, may chemically and physically modify the wound surface, leading to enhanced wound-healing effects. These findings suggest that the combination of CP and water can potentially have synergistic effects on wound healing processes.

The study by Nakajima et al. (144) demonstrated that the combination of plasma treatment and pouring water had a significant impact on enhancing the strength of myofibroblasts. This suggests a correlation between the reduction of wound size and an increase in the number of myofibroblasts. Furthermore, the combined treatment of plasma and water pouring was more effective in promoting wound contraction compared to plasma treatment alone.

In a study conducted on New Zealand white rabbits, Alhabshan et al. (145) analyzed the effects of CAP on wound healing after corneal epithelial and basement membrane ablation. The results indicated that CAP did not hinder the rate of wound closure, induce increased inflammation, or adversely affect corneal wound healing. These findings highlight the potential of CP as a beneficial treatment for wound healing, and further research on the impact of CP on wound healing in animals can be explored in Table 3.

**Table 3:** Studies related to the effect of cold plasma on animal skin.

Disease or pathogen	Target animal	CP type	Results	Reference
Intact skin	Murine	CP	The disaggregation of cells in the stratum corneum (SC) is increased, and tissue oxygenation is also increased	(115)
Cutaneous wound	Rat	CP	It accelerates blood coagulation and enhances the healing process of cutaneous wounds	(146)
Intact skin	Dog	CAMP	CAMP treatment was well-tolerated and did not cause significant changes in the biophysical parameters of dog skin	(141)
Cutaneous wound	Murine	APPJ	Significantly accelerated wound re-epithelialization was demonstrated between days three and nine	(143)
Cutaneous wound	Mice	APPJ	The treatment of plasma with dropped water had a more significant effect than plasma treatment alone	(144)
Cutaneous wound	Rat	CAP	The CAP group showed a decrease in the size of the wound area and a significant increase in the protein level of smooth muscle actin	(138)
Cutaneous wound	Mouse	CAP	CAP treatment induces the expression of key genes involved in the wound healing response, including IL-6, IL-8, MCP-1, TGF-ß1, TGF-ß2, collagen type I, and alpha-SMA	(137)
Keratinocytes	Canine	CAMP	Gene expression: cell cycle, proliferation, angiogenesis, adhesion, wound healing	(128)
*S. aureus*	Rabbit	APPJ	Regulation of cytokine secretion, limited inflammation, promotion of wound healing, and bacteria inactivation	(142)
*E. coli*	Canine	CAMP	CAAP treatment achieved satisfactory decontamination rates (98.9%–99.9%) for all bacteria species *in vitro*. The decontamination rate was primarily determined by the initial bacterial concentration and treatment time	(72)
*S. aureus*				
*Pasteurella multocida*				

CP, cold plasma; CAMP, cold atmospheric microwave plasma; APPJ, atmospheric pressure plasma jet; CAP, cold atmospheric plasma.

## 8. Advantages and limitations of cold plasma for veterinary bacterial diseases

In veterinary medicine, there is growing interest in utilizing CP, also known as non-thermal plasma, as a promising treatment option for bacterial diseases. This innovative technology creates a plasma field that generates active species such as reactive oxygen and nitrogen species (RONS), known for their antimicrobial properties. The use of CP in animal healthcare brings various advantages, including its ability to combat bacterial infections effectively. However, there are also certain limitations and restrictions associated with its application. Despite these restrictions, CP holds great potential as an alternative treatment for bacterial diseases in veterinary medicine (59).

Advantages: safety: CP is a non-thermal process that poses no risk of heat generation, which makes it a safe option for treating animals. Unlike traditional thermal plasma, it does not harm tissues by causing thermal injury, therefore it can be used in sensitive areas such as the eye or ear. Efficacy: CP has exhibited antimicrobial properties against a broad spectrum of bacteria, including resistant strains. In veterinary medicine, it has been successfully employed to treat bacterial infections in animals, including skin and wound infections. Versatility: CP can be administered in various forms, including sprays and topical applications, rendering it a versatile treatment option for different kinds of bacterial infections. Environmental sustainability: CP does not produce hazardous waste; thus, it is an environmentally friendly alternative to traditional antibiotics. Limitations: cost: the equipment required for CP treatment can be expensive, making it a cost-prohibitive option for some veterinary practices. Lack of standardization: there is currently a lack of standardization in the field of CP treatment, making it difficult to compare the efficacy of different devices. Limited research: although CP has shown promise as a treatment option for bacterial infections in animals, further research is needed to fully understand its mechanism of action and efficacy. Regulatory approval: CP is currently not approved by regulatory agencies for the treatment of bacterial infections in animals, making it difficult for veterinary practices to use it in a clinical setting.

In conclusion, CP shows promise as a treatment option for bacterial diseases in veterinary medicine. However, more research is necessary to gain a comprehensive understanding of its mechanisms of action, effectiveness, and safety. Furthermore, the high cost of equipment and the absence of regulatory approval may restrict its widespread adoption in the field of veterinary medicine. Despite these challenges, continued exploration and development of CP technology could potentially overcome these limitations and contribute to improved treatment options for bacterial diseases in animals.

### 8.1. Future directions for cold plasma research in veterinary bacteriology

Although the use of CP has demonstrated promising outcomes in managing a range of bacterial diseases in veterinary medicine, there is still a lot to be explored in this domain. To further enhance the use of CP in veterinary bacteriology, various areas of research must be considered. One of the crucial areas for future exploration is the development of new plasma sources. At present, most CP devices are based on high-voltage discharge systems, which can be intricate and costly to operate. There is a requirement for the development of more compact and cost-efficient plasma sources that can be easily integrated into veterinary clinics. Furthermore, research is needed to evaluate the effectiveness of these new plasma sources in controlling various bacterial infections (147). Optimization: the optimization of treatment protocols is a crucial field of study that requires attention. At present, information is scarce regarding the ideal conditions for employing CP for managing bacterial infections in animals. It is necessary to conduct research to determine the most efficient plasma parameters, including treatment duration, power density, and exposure range, for various bacterial strains and hosts. This initiative will aid in enhancing the effectiveness and safety of CP treatments in veterinary medicine (148). Evaluation of the mechanisms of action: the precise means by which CP eradicates bacteria is yet to be fully comprehended. Additional research is necessary to assess the mechanisms of action of CP and its interactions with various bacterial species and hosts. This knowledge will be essential in the creation of novel plasma sources and treatment protocols that are tailored to manage specific bacterial infections (149). Although several studies have deemed CP safe and non-toxic, further research is required to evaluate its safety and toxicity in animals. This will enable us to determine any potential side effects of plasma treatments and verify their safety for use in veterinary medicine. To summarize, while CP exhibits potential as a tool for controlling bacterial infections in veterinary medicine, extensive research and development are still necessary. Key areas for future investigation in this field include the development of new plasma sources, optimization of treatment protocols, evaluation of mechanisms of action, and assessment of safety and toxicity (125).

## 9. Conclusion

In conclusion, the use of CP in veterinary medicine for treating bacterial diseases is a promising and emerging field of research. CP has demonstrated significant potential in managing various bacterial infections in veterinary medicine, including respiratory infections, skin infections, and wound infections (36). One of the most notable advantages of implementing CP is its capability to eradicate a wide spectrum of bacteria, which includes strains that are resistant to antibiotics. Moreover, it can boost the immune system and expedite the process of wound healing. Nevertheless, CP therapy in veterinary medicine has certain limitations. Ensuring consistent and uniform exposure of the affected area to the plasma can pose a challenge and may affect its efficacy. Additionally, more research needs to be conducted to establish optimal treatment conditions, such as exposure time and plasma density, for different types of bacterial infections. Although the use of CP in veterinary medicine holds great potential, further investigation is imperative to fully comprehend its advantages and drawbacks. Future studies should concentrate on optimizing treatment protocols and assessing the long-term consequences of CP therapy on both bacterial infections and animal health.

## Author contributions

PM and NF wrote the first draft. AG revised the manuscript. All authors contributed to the article and approved the submitted version.

## Funding

This work is based upon research funded by Iran National Science Foundation (INSF) under Project No. 4015898.

## Conflict of interest

The authors declare that the research was conducted in the absence of any commercial or financial relationships that could be construed as a potential conflict of interest.

## Publisher’s note

All claims expressed in this article are solely those of the authors and do not necessarily represent those of their affiliated organizations, or those of the publisher, the editors and the reviewers. Any product that may be evaluated in this article, or claim that may be made by its manufacturer, is not guaranteed or endorsed by the publisher.
